# Benefits of Vitamins in the Treatment of Parkinson's Disease

**DOI:** 10.1155/2019/9426867

**Published:** 2019-02-20

**Authors:** Xiuzhen Zhao, Ming Zhang, Chunxiao Li, Xue Jiang, Yana Su, Ying Zhang

**Affiliations:** ^1^Department of Neurology and Neuroscience Center, First Hospital of Jilin University, Xinmin Street No. 71, Changchun 130000, China; ^2^Department of Pharmacology, College of Basic Medical Sciences, Jilin University, 126 Xin Min Street, Changchun, Jilin 130021, China; ^3^Department of Neurology and Neuroscience Center, The Affiliated Hospital of Qingdao University, Qingdao, Shandong 266000, China

## Abstract

Parkinson's disease (PD) is the second most common neurodegenerative disease in the elderly, which is clinically characterized by bradykinesia, resting tremor, abnormal posture balance, and hypermyotonia. Currently, the pathogenic mechanism of PD remains unclear. Numerous clinical studies as well as animal and cell experiments have found a certain relationship between the vitamin family and PD. The antioxidant properties of vitamins and their biological functions of regulating gene expression may be beneficial for the treatment of PD. Current clinical evidence indicates that proper supplementation of various vitamins can reduce the incidence of PD in the general population and improve the clinical symptoms of patients with PD; nevertheless, the safety of regular vitamin supplements still needs to be highlighted. Vitamin supplementation may be an effective adjuvant treatment for PD. In this review, we summarized the biological correlations between vitamins and PD as well as the underlying pathophysiological mechanisms. Additionally, we elaborated the therapeutic potentials of vitamins for PD.

## 1. Introduction

Parkinson's disease (PD) is the second most common neurodegenerative disorder following Alzheimer's disease. Clinically, PD is characterized by resting tremor, hypermyotonia, postural instability, and bradykinesia [1]. Additionally, patients with PD can also manifest with nonmotor symptoms, such as cognitive decline, olfactory dysfunction, constipation, sleep disorders, and autonomic symptoms [2], and these nonmotor symptoms usually occur prior to the onset of motor symptoms [3]. PD severely affects the quality of life of the individual with the disease and also creates a great burden on the caregivers. The typical pathological hallmark of PD is degeneration of dopaminergic neurons in the substantia nigra pars compacta (SNpc) and eosinophilic inclusion bodies (Lewy bodies) in the remaining neurons, which is the major contributor to the deficiency of dopamine in the basal ganglia [4, 5]. The exact pathogenetic mechanisms of PD is not yet fully understood. Current theories regard PD as a multifactorial disease, involving various genetic and environmental factors, among which mitochondrial dysfunction and oxidative stress play an important role in the pathogenesis and development of PD [6, 7]. The treatment for PD is challenging, and the existing therapeutic strategies can only relieve clinical symptoms but fail to control the progression of PD.

Vitamins are natural bioactive products with antioxidant properties, which are necessities for maintaining the normal functions of human organisms. Essential vitamins cannot be endogenously synthesized in the organism and therefore must be obtained through the diet. Clinically, vitamin deficiency is quite common, especially in infants and elderly. Vitamins are generally divided into fat-soluble variants (vitamins A, D, E, and K) and water-soluble variants (vitamins B and C). The former mainly bind to cellular nuclear receptors and affect the expression of specific genes [8]. The latter mainly constitute a cofactor for the enzyme, affecting the enzymatic activity [9].

Numerous clinical studies as well as animal and cell experiments have found a certain relationship between the vitamin family and PD [10]. The antioxidant properties of vitamins and their biological functions of regulating gene expression may be beneficial for the treatment of PD. Current clinical evidence indicates that proper supplementation of various vitamins can reduce the incidence of PD in the general population and improve the clinical symptoms of patients with PD; nevertheless, the safety of regular vitamin supplements still needs to be highlighted. Vitamin supplementation may represent an effective adjuvant treatment for PD. In this review, we summarized the biological correlations between vitamins and PD as well as the underlying pathophysiological mechanisms. Additionally, we elaborated the therapeutic potentials of vitamins for PD.

## 2. The Pathogenesis of Oxidative Stress in PD

Oxidative stress refers to the imbalance between the oxidation system and antioxidant system, resulting in excessive accumulation of oxidative substances, such as reactive oxygen species (ROS) and reactive nitrogen species (RNS) [11]. ROS include superoxide anion radical (O_2_^−^), hydroxyl radical (OH^−^), and hydrogen peroxide (H_2_O_2_); RNS include nitric oxide (NO), nitrogen dioxide (NO_2_), and peroxynitrite (ONOO^−^). The antioxidant system mainly consists of two subtypes: (1) enzymatic antioxidant system, including superoxide dismutase (SOD), catalase (CAT), and glutathione peroxidase (GSH-Px), and (2) nonenzymatic antioxidant system, including vitamin C, vitamin E, glutathione, melatonin, alpha-lipoic acid, carotenoids, and trace elements copper, zinc, and selenium.

Oxidative stress plays an important physiological role in the organism. For example, phagocytic cells kill pathogenic microorganisms, participate in detoxification and enzymatic reactions, and synthesize some essential biologically active substances. Meanwhile, it can as well cause damage to the body, such as cell membrane destruction, protein denaturation, and nucleic acid changes.

There is increasing evidence that oxidative stress represents a pathophysiological characteristic of PD, and the production of reactive oxygen species can result in neuronal death [12, 13]. The mitochondrial respiratory chain is regarded as the major source of ROS [14]. Additionally, previous studies have found that mitochondrial dysfunction exists in the substantia nigra of patients with PD [15]. Reduced glutathione (GSH) can enhance the production of ROS and RNS [16], and oxidation of dopamine and dopamine metabolites such as 3,4-dihydroxyphenylacetic acid (DOPAC) can inhibit the activity of complex I [17]. These findings indicate that the downstream metabolites of dopamine may make dopamine neurons more susceptible. Moreover, iron accumulation in the substantia nigra is common in patients with PD, leading to overproduction of hydrogen peroxide and molecular oxygen in the Fenton reaction from Fe^2+^ to Fe^3+^; hydrogen peroxide generates a highly toxic hydroxyl radical through the Haber-Weiss reaction in the presence of Fe^2+^, which causes severe oxidative damage to the cellular components [18]. From the above, the oxidant stress is closely associated with the pathogenesis of PD. Oxidative stress can cause neuronal loss through some underlying intracellular damage, such as protein aggregation, mitochondrial dysfunction, and DNA rupture. Therefore, antioxidant damage has become a potential target for the treatment of PD.

## 3. Vitamin B and PD

The B family of vitamins is water-soluble, which includes thiamine (vitamin B_1_), riboflavin (vitamin B_2_), niacin (vitamin B_3_), pantothenic acid (vitamin B_5_), pyridoxine (vitamin B_6_), biotin (vitamin B_7_), folate (vitamin B_9_), and cobalamin (vitamin B_12_) [19]. These vitamins play an important role as enzyme cofactors in multiple biochemical pathways in all tissues, such as regulating metabolism, improving the function of the immune system and nervous system, and promoting cell growth and division [20].

Almost all of these B family vitamins are essential variants dependent on diet supply, except niacin which can also be synthesized from tryptophan. Vitamin B deficiencies are frequent in the children, elderly, vegetarians, pregnant women, and patients with gastrointestinal diseases. Recently, the association with vitamin B and PD is getting more and more attention. Herein, we use vitamin B_3_ as a representative to discuss the relationship between vitamin B and PD.

### 3.1. Vitamin B_3_

Nicotinamide is the active form of niacin, and it is the precursor of coenzymes NADH and NADPH, which are essential for over 200 enzymatic reactions in the organism, especially the production of adenosine triphosphate (ATP). Meat, fish, and wheat are generally rich in nicotinamide, while vegetables have a low nicotinamide content [21]. Deficiency of nicotinamide/niacin can lead to pellagra, causing dermatitis, diarrhea, and depression [22]. Nicotinamide has neuroprotective and antioxidant functions at low doses but exhibits neurotoxicity, especially dopaminergic toxicity, at high doses [23]. Fukushima also suggests that excessive nicotinamide is related to the development of PD [24]; excessive nicotinamide can induce overproduction of 1-methylnicotinamide (MNA), which is increased in patients with PD [25]. In an *in vitro* study, Griffin et al. found that low-dose nicotinamide (10 mM) has a significant effect on inducing differentiation from embryonic stem cells into neurons; however, higher doses (>20 mM) of nicotinamide induce cytotoxicity and cell death [26]. The definitive protective dose of vitamin B_3_ still needs further researches.

### 3.2. Possible Neuroprotective Mechanisms of Vitamin B_3_ in PD

Firstly, numerous studies have demonstrated that mitochondrial dysfunction and cellular energy failure are pathophysiological features of PD. Nicotinamide participates in the biosynthesis of nicotinamide adenine dinucleotide (NAD; oxidized form: NAD^+^; reduced form: NADH) via various metabolic pathways [27]. NADH is an essential cofactor assisting the tetrahydrobiopterin functioning in tyrosine hydroxylase, which can hydroxylate tyrosine and produce dopamine; NADH deficiency is common in PD [28].

Secondly, NADH is indispensable for the physiological function of mitochondrial complex I in ATP synthesis, and the corresponding dysfunction is involved in PD patients and animal models [15, 29, 30]. Nicotinamide mononucleotide (NMN) constitutes one of the key precursors of NAD^+^. In previous *in vitro* studies, the scholars have established a cellular model of PD using rotenone-treated PC12 cells, and they found the NMN (0.1 mM or 1 mM) treatment was associated with a significantly higher survival rate in the rotenone-treated (0.5 *μ*m) PC12 cells. NMN is assumed to enhance the intracellular levels of NAD^+^ and ATP in the cellular model of PD [31].

In addition, nicotinamide can act a neuroprotective role by inhibiting the oxidative stress. In 1-methyl-4-phenyl-1,2,3,6-tetrahydropyridine- (MPTP-) induced mouse models of PD, nicotinamide (500 mg/kg) was injected before subacute (30 mg/kg/d for 5 days) MPTP administration. This study showed that cotreatment with MPTP and nicotinamide significantly improved the locomotor activity compared to single-agent treatment with MPTP. Nicotinamide administration significantly attenuated the MPTP-induced dopamine depletion (47.11 ± 21.06 vs. 12.77 ± 8.06). Meanwhile, nicotinamide pretreatment markedly inhibited MPTP-induced lactate dehydrogenase (LDH) and NOS activities, which prevented the oxidative stress and alleviated the oxidative damage.

Sirtuins (SIRTs) are NAD^+^-dependent protein deacetylases involved in vital biological processes [32]. Recently, sirtuin 5 (SIRT5) has received considerable attention. Liu et al. investigated the role of SIRT5 in MPTP-induced mouse PD models [33]. They found that deletion of SIRT5 exacerbated motor deficits, nigrostriatal dopaminergic degeneration in the compact part of substantia nigra (SNc), and mitochondrial antioxidant activities in the PD models. These findings provide new insight into the therapeutic strategies for PD. However, the protective effects of nicotinamide are still controversial, and further researches are needed to clarify the biological function of vitamin B_3_ in PD.

### 3.3. Clinical Studies regarding Vitamin B_3_ in PD

Current existing clinical studies have shown that a high-niacin diet can reduce the risk of PD [34, 35]. A previous case report also demonstrated that oral niacin (500 mg twice daily for three months) significantly improved rigidity and bradykinesia in a patient with idiopathic PD, though the original purpose was to treat hypertriglyceridemia; after the cessation of oral niacin due to obvious adverse effects (unacceptable nightmares and skin rash), the symptoms of rigidity and bradykinesia relapsed [36]. However, other studies failed to notice the remarkable clinical efficacy [37, 38]. Therefore, more clinical observations are warranted to verify the efficacy as well as side effects of niacin in PD.

## 4. Vitamin C and PD

Vitamin C (ascorbic acid) is another water-soluble, essential vitamin, which is widely distributed in various tissues. This nutriment is abundant in vegetables, fresh fruits, and animal livers. Vitamin C contains two molecular subforms in organisms: the reduced form (ascorbic acid (AA)) and the oxidized form (dehydroascorbic acid (DHA)). Deficiency of vitamin C is common, especially in children and elderly. A long-term lack of vitamin C can cause scurvy. Vitamin C is very important for the physiological function of the nervous system and the antioxidant function by inhibiting oxidative stress, reducing lipid peroxidation, and scavenging free radicals [39]. Moreover, it is also involved in many nonoxidative stress processes, such as synthesis of collagen, cholesterol, carnitine, catecholamines, amino acids, and some peptide hormones [40, 41].

Dopamine metabolism can produce oxidative stress products, which in return induce accumulation of abnormal proteins in PD [42]. Vitamin C has potentials for the treatment of PD considering the following reasons. Firstly, vitamin C is mainly distributed in areas that are rich in neurons [43, 44]. Secondly, vitamin C can be transported to the brain by SVCT2 (vitamin C transporter type 2) [45], and DHA can be transported to the brain by GLUT1 (glucose transporter type 1) and GLUT3 (glucose transporter type 3) [46].

### 4.1. Possible Neuroprotective Mechanisms of Vitamin C in PD

There is evidence that ascorbic acid can protect against both levodopa toxicity and the MPTP neurotoxicity [47, 48]. Vitamin C can increase the production of dihydroxyphenylalanine (DOPA). Seitz et al. noted overproduction of DOPA in a dose-dependent manner after incubation of the human neuroblastoma cell line SK-N-SH with ascorbic acid (100-500 mM) for 2 hours. Additionally, the gene expression of tyrosine hydroxylase increased threefold after incubation with ascorbic acid (200 mM) for 5days. The scholars speculated that ascorbic acid may be effective in the treatment of early-stage PD [49].

Vitamin C can improve the absorption of levodopa in elderly PD patients with a poor levodopa bioavailability [50]. Previous studies showed that ascorbic acid can reduce the levodopa dosage under the premise of equal efficacy [51]. Combination of anti-PD drugs and vitamin C may be more effective for alleviating the symptoms of PD.

Vitamin C is essential for the brain development. A study showed that ascorbic acid treatment can promote a 10-fold increase of dopaminergic differentiation in CNS precursor cells derived from the E12 rat mesencephalon [52]. Soon after, another *in vitro* study also reported that AA can stimulate the CNS precursor cells differentiating into CNS neurons and glia [53]. Recently, He et al. proposed that vitamin C can greatly enhance the embryonic midbrain neural stem cells differentiating into midbrain dopaminergic neurons in vitro. Vitamin C induces the gain of 5-hydroxymethylcytosine (5HMC) and loss of H3K27m3 in dopaminergic phenotype gene promoters, which are catalysed by ten-eleven translocation 1 methylcytosine dioxygenase 1 (TET1) and histone H3K27 demethylase (JMJD3), respectively. However, subsequent TET1 and JMJD3 knockdown/inhibition experiments did not show this effect of vitamin C, and the epigenetic role of vitamin C may be associated with the midbrain dopaminergic neuron development [54, 55].

### 4.2. Clinical Studies regarding Vitamin C in PD

Although vitamin C has many potential positive effects on PD, the serum level of vitamin C in patients with PD remains controversial [56, 57]. Noteworthily, the vitamin C level in lymphocytes has been found significantly lower in patients with severe PD [58]. Theoretically, vitamin C supplementation may be beneficial for the treatment of PD. A cohort study involving 1036 patients with PD supported this hypothesis, which found that dietary vitamin C intake significantly reduced the risk of PD, but this effect is invalid for a 4-year-lag analysis [59]. Controversially, many studies did not support that vitamin C supplementation can reduce the risk of PD [10, 60, 61]. We speculate this contradiction may be related to the timing of vitamin application.

## 5. Vitamin E and PD

Vitamin E is a fat-soluble vitamin with high antioxidant properties. Natural vitamin E includes two subgroups: tocopherols and tocotrienols; and they can further be divided into four lipophilic molecules, respectively: *α*-, *β*-, *γ*-, and *δ*-tocopherol (*α*T, *β*T, *γ*T, and *δ*T) and *α*-, *β*-, *γ*-, and *δ*-tocotrienol (*α*TE, *β*TE, *γ*TE, and *δ*TE). The major difference between tocopherols and tocotrienols is the side chain. Tocopherols have a saturated phytyl tail, while tocotrienols possess an unsaturated isoprenoid side chain [62]. Because of this unsaturated side chain, the tocotrienol is superior to the tocopherol as an antioxidant by increasing the molecular mobility through lipid membranes and by accepting electrons readily. Overt vitamin E deficiency is relatively rare, mainly in infants and premature babies.

In addition to its potent antioxidant capacity, vitamin E is involved in many physiological processes such as immune function [63], cognitive function, physical performance [64, 65], and regulation of gene expression. In humans, deficiency of vitamin E is clinically characterized by peripheral neuropathy, ataxia, and anemia [66, 67].

### 5.1. Possible Neuroprotective Mechanisms of Vitamin E in PD

Unilateral 6-hydroxydopamine (6-OHDA) injections into the striatum can cause circling behaviours and biochemical abnormalities in rats. Cadet et al. found that pretreatment with either D-alpha-tocopherol or all-racemic-alpha-tocopherol significantly attenuated these pathological changes [68]. Roghani and Behzadi [69] and Sharma and Nehru [70] also demonstrated the similar phenomenon in 6-OHDA-induced PD models and in rotenone-induced PD models, respectively. However, some studies have shown that vitamin E did not completely protect dopaminergic neurons from MPTP-mediated damage in PD models [71, 72]. The protective effects of vitamin E may be achieved through preventing oxidative stress in cells and inhibiting apoptosis. Moreover, one study has found that tocotrienol participates not only in antioxidant stress but also in estrogen receptor beta (ER*β*) signal transduction [73]. Then, Nakaso's team demonstrated a protective effect of vitamin E via this signaling pathway. Firstly, they reported that *γ*-tocotrienol/*δ*-tocotrienol exerts neuroprotective effects through the ER*β*-PI3K/Akt signaling pathways in SH-SY5Y cells by resisting 1-methyl-4-phenylpyridiniumion- (MPP^+^-) induced toxicity [74]. Secondly, they verified this mechanism in a mouse model of PD. Meanwhile, they found *δ*-tocotrienol administration can reduce the loss of dopaminergic neurons in the substantia nigra and ER inhibitors can attenuate this neuroprotective effect [75]. These findings indicate vitamin E may be potential therapeutic agents for PD.

### 5.2. Clinical Studies regarding Vitamin E in PD

The DATATOP (Deprenyl and Tocopherol Antioxidative Therapy of Parkinsonism) experiment is a multicentre-controlled clinical trial to investigate the long-term efficacy of treatment with deprenyl and/or copherol (vitamin E) and to explore whether it is possible to extend the time before the application of levodopa treatment. At 28 US and Canadian sites, 800 eligible patients with untreated early-stage PD were enrolled in DATATOP and randomized to four groups: (1) deprenyl 10 mg/d, (2) copherol 2000 IU/d, (3) placebo-controlled, and (4) deprenyl 10 mg/d and copherol 2000 IU/d. Deprenyl can delay the development of functional disorders, delay the application of levodopa, and improve motor symptoms, but vitamin E is disappointing [76]. Similarly, another two population-based studies also did not find the association between vitamin E intake and risk of PD [10, 77].

However, a large community-based study showed that high intake of dietary vitamin E (10 mg/day) may reduce the occurrence of PD [78]. Another pilot trail suggests that long-term treatment with vitamin E may delay the use of levodopa in patients with PD [79]. Further research is needed to verify these results.

Although there seems to be no difference in the level of alpha-tocopherol (vitamin E) in serum, cerebrospinal fluid, and brains between PD and normal controls [80–82], there is evidence showing that high-dose vitamin E (2000 IU/day) can significantly elevate the vitamin E level in cerebrospinal fluid [83]. At present, the protective mechanism of vitamin E in PD is still unclear and may be related to the strong antioxidant effect of vitamin E. Further research is needed to determine whether vitamin E can be used as a potential treatment for PD.

## 6. Vitamin D and PD

Vitamin D, a steroid hormone, is crucial for calcium homeostasis and skeletal health. This nutriment mainly includes two forms: vitamin D_2_ and vitamin D_3_; the latter is endogenously produced when skin is exposed to UV-B rays from the sun. Both of the above forms are inactive, and they are transformed into the active form 1,25-dihydroxy vitamin D3 (1,25-(OH)_2_-D_3_) after being hydroxylated twice [84, 85]. 1,25-(OH)_2_-D_3_ were secreted into the blood system by the kidney, having a direct effect on gene regulation by binding to the nuclear vitamin D receptor (VDR) [86, 87]. Vitamin D deficiency is prevalent at all ages, especially in elderly. Vitamin D not only regulates the calcium homeostasis and skeletal health but also regulates the physiological and pathological processes, such as cell proliferation and differentiation, immunomodulatory, and antioxidative stress [88–90]. Children with a lack of vitamin D may suffer from rickets, and adults may develop osteomalacia. Additionally, vitamin D deficiency is also associated with cardiovascular diseases, muscle weakness, diabetes mellitus, cancers, and multiple sclerosis [91]. The relationship between vitamin D and PD has gradually attracted attention [92].

### 6.1. Possible Neuroprotective Mechanisms of Vitamin D in PD

VDR belongs to the intranuclear receptor superfamily, composing of eight coding exons and three alternative 5′ noncoding exons, spanning over 105 kb, on chromosome 12 [93]. The most widely studied biallelic polymorphic sites are BsmI, TaqI, ApaI, and FokI. Substantial researches have been carried out to explore the relationship between these allelic variations and PD. Kim et al. detected VDR gene BsmI polymorphisms in over 300 Korean individuals (85 PD and 231 controls). The frequency of VDR genotype *bb* was significantly increased in the PD patients (84.7%) than that in the controls (72.7%). The *bb* genotype was more common in PD patients with postural instability and gait difficulty than in the PD patients with tremor (94.3% vs. 75.6%) [94]. A meta-analysis showed that VDR BsmI and FokI polymorphisms were associated with the risk of PD [95], and VDR FokI genotype was associated with the severity and cognitive decline of PD [96, 97]. Muscular and motor impairments, which can seriously affect the motor behaviour, were found in the VDR-knockout mice [98], indicating that vitamin D may be involved in the pathogenesis of PD.

Glial cell line-derived neurotrophic factor (GDNF) is a protein that is essential for the maintenance and survival of dopaminergic neurons and can inhibit microglial activation [99]. Many animal studies showed that 1,25-(OH)_2_-D_3_ could enhance the endogenous GDNF expression in vitro and in vivo and inhibit the glial cell activation to protect dopaminergic neurons from immune inflammation [100–102].

Vitamin D_3_ can protect dopaminergic neurons against 6-hydroxydopamine-mediated neurotoxicity and improve the motor performance in the 6-hydroxydopamine-induced PD rat [103]. It may be related to vitamin D's properties of inhibiting oxidative stress and decreasing the production of reactive oxygen species and free radicals [104]. In addition, endothelial dysfunction may be associated with low vitamin D levels in patients with PD [105]. The definitive correlations between vitamin D and PD require more researches.

### 6.2. Clinical Studies regarding Vitamin D in PD

Substantial epidemiological and clinical studies suggest that vitamin D has a positive effect on PD. In a cohort study, over 7000 Finnish's serum samples were collected for measuring the 25-hydroxy vitamin D level, and meanwhile, the occurrence of PD was instigated over a 30-year follow-up period. The results showed that individuals with higher serum vitamin D concentrations had a lower risk of PD [106]. Evatt et al. also noted consistent findings [107].

As mentioned above, vitamin D_3_ can be endogenously synthesized upon sunlight exposure in the skin. In a large case-control study of Danish men, involving 3819 PD patients and 19,282 controls, the scholars proposed that men working outdoors have a lower risk of PD [108]. Another nationwide ecologic study in France also suggests that vitamin D levels are negatively correlated with the risk of PD, but this result needs taking ages into account [109]. Wang et al. not only demonstrated a positive correlation between serum 25-hydroxy vitamin D and sunlight exposure but also noted that lower serum levels of 25-hydroxy vitamin D and sunlight exposure can increase the risk of PD [110].

Furthermore, PD patients with lower 25-hydroxy vitamin D levels may exhibit more severe symptoms compared with normal controls [111, 112]. Unsurprisingly, a randomized, double-blind, placebo-controlled trial found that vitamin D_3_ supplementation (1200 IU/day for 12 months) significantly prevented the deterioration of PD [113].

## 7. Conclusion

In summary, vitamins may play a protective role in PD. Among the fat-soluble vitamins, we briefly summarized the effects of vitamin E and vitamin D. At present, although many studies have shown that vitamin E supplementation can reduce the risk of Parkinson's disease (Table 1), the DATATOP study has showed that vitamin E supplementation is ineffective in Parkinson's disease (Table 2). Many noninterventional studies found that the high levels of serum vitamin D can reduce the risk of PD (Table 1), and several clinical intervention trials also proposed that vitamin D supplementation can attenuate the deterioration of the Parkinson's disease and reduce the occurrence of fractures in patients with PD (Table 2). Among the water-soluble vitamins, we elaborated the functions of vitamin B_3_ and vitamin C. There is still a paucity of clinical evidence for determining the pros and cons of vitamin B_3_ in PD (Table 2). Vitamin C is vital to the human organism, and it can improve levodopa absorption in elderly PD patients (Table 2); current epidemiological evidence is still insufficient to establish a correlation between the serum level or dietary intakes of vitamin C and the risk of PD (Table 1). Although there have been many researches on the relationship between vitamins and PD (Tables 1–3), there is still lack of a clinical intervention trial explicitly confirming that vitamin supplementation can reduce the incidence of PD and prevent the progression of the disease. Moreover, the individual physical and chemical properties, absorption rate, and bioavailability of vitamins may affect the efficacy. Further studies are still needed to clarify the potentials of vitamins for the treatment of PD.

## Figures and Tables

**Table 1:** The other clinical study of vitamins and Parkinson's disease.

Vitamins	Authors	Type of study	Patients/controls	Conclusions
Vitamin B_3_	Abbott et al. [37]	A Honolulu-Asia Aging Study in Japanese-American	Total 8006 and observed 137 PD	Niacin has no obvious relationship with clinical PD
	Johnson et al. [38]	A case-control study in US	126/432	Niacin has no relationship with PD
	Fall et al. [34]	A case-control study in Sweden	113/263	High-niacin diet can reduce the risk of PD
	Hellenbrand et al. [35]	A case-control study in German	342/342	PD patients with lower intake of niacin than controls
Vitamin C	Yang et al. [61]	A prospective study in Sweden	Total 84,774 and observed 1329 PD cases	Intake of vitamin C has a negative correlation with PD risk in women at borderline significance (*P* = 0.04)
	Hughes et al. [59]	A prospective study in American	Total 129,422 and observed 1036 PD cases	Intake of vitamin C has no relationship with PD risk
	Ide et al. [58]	A case-case study in Japan	62 PD	The severe PD patients with significantly lower lymphocyte vitamin C levels (*P* < 0.01)
	Miyake et al. [60]	A case-control study in Japan	249/368	Intake of vitamin C has no relationship with PD risk
	Zhang et al. [10]	A prospective study in US	Total 124,221 and observed 371 PD cases	Intake of vitamin C has no relationship with PD risk
	Férnandez-Calle et al. [56]	A case-control study in Spain	63/63	Vitamin C has no relationship with PD
	King et al. [127]	A case-control study in United States	27/16	Vitamin C was higher in PD groups
Vitamin E	Yang et al. [61]	A prospective study in Sweden	Total 84,774 and observed 1329 PD cases	Dietary intake of vitamin E has negative correlation with the incidence of PD in women (*P* = 0.02)
	Hughes et al. [59]	A prospective study in American	Total 129,422 and observed 1036 PD cases	Vitamin E has no relationship with PD risk
	Miyake et al. [60]	A case-control study in Japan	249/368	Vitamin E significantly reduced the risk of PD
	Zhang et al. [10]	A prospective study in US	Total 124,221 and observed 371 PD cases	Intaking foods containing more vitamin E can reduce the risk of Parkinson's disease
	Molina et al. [82]	A case-control study in Spain	34/47	CSF and serum vitamin E levels have no difference between two groups
	de Rijk et al. [78]	A cross-sectional study in Netherlands	5342 individuals including 31 PD cases	Intaking 10 mg dietary vitamin E daily may reduce the risk of PD
	Logroscino et al. [77]	A case-control study in USA	110/287	Vitamins A, C, and E were not associated with PD
	Férnandez-Calle et al. [80]	A case-control study in Spain	42/42	Serum levels of alpha-tocopherol (vitamin E) have no difference between two groups
Vitamin D	Kim et al. [128]	A prospective, observational study in Korea	39 PD cases	The level of vitamin D might impact the olfactory dysfunction in PD
	Sleeman et al. [112]	A prospective observational study in England	145/94	Serum 25(OH)D concentrations are often lower in PD patients than controls and relate to the severity of motor symptoms
	Wang et al. [110]	A case-control study in China	201/199	The serum 25(OH)D and sunlight exposure inversely correlated with PD occurrence
	Shrestha et al. [129]	A prospective study in USA	Total 12,762 participants and observed 67 PD cases	This study did not suggest that the vitamin D can reduce the risk of PD
	Liu and Zhang [111]	A case-control study in China	229/120	The 25(OH) D levels may be inversely associated with the PD severity
	Lin et al. [130]	A case-control study in China	700/792	They have not found the associations between the genetic variants of VDR and PD occurrence
	Zhu et al. [131]	A case-control study in China	209/210	Outdoor activity and total vitamin D intake may reduce the risk of PD
	Petersen et al. [132]	A case-control study in Denmark	121/235	They have not found the association between PD and vitamin D polymorphisms and/or 25(OH)D levels
	Török et al. [133]	A case-control study in Hungary	100/109	It showed the association between the FokI C allele and PD
	Lv et al. [134]	A case-control study in China	483/498	The study did not support the relationship between VDR gene and PD
	Peterson et al. [135]	A cross-sectional, observational study in USA	40 PD	Serum vitamin D levels are inversely related to the severity of Parkinson's disease and play an important role in balance of PD
	Suzuki et al. [96]	A prospective cohort study in Japan	137 PD	The 25-hydroxyvitamin D levels and the vitamin D receptor FokI CC genotype may be associated with the severity of the PD
	Kenborg et al. [108]	A case-control study in Denmark	3819/19,282	This study supports that working outdoors can reduce the risk of PD
	Evatt et al. [136]	A survey study in USA	199 PD (from DATATOP)	Vitamin D insufficiency is very common in early PD patients
	Miyake et al. [137]	A case-control study in Japan	249/368	The study showed that vitamin D was not related to the PD
	Knekt et al. [106]	The Mini-Finland Health Survey in Finland	Total 3173 and observed 50 PD cases	The serum vitamin D concentrations were inversely correlated with the risk of PD
	Kim et al. [94]	A case-control study in Korea	85/231	Vitamin D receptor gene polymorphism was associated with the PD

**Table 2:** The clinical intervention trial of vitamins and Parkinson's disease.

Vitamins	Authors	Patients	Treatment	Conclusions
Vitamin C	Nagayama et al. [50]	67 elderly PD patients	200 mg ascorbic acid	Ascorbic acid can improve levodopa absorption in elderly PD patients
Vitamin E	Parkinson Study Group (DATATOP study) [76]	800 untreated and early PD patients	Deprenyl 10 mg/d and/or tocopherol (vitamin E) 2000 IU/d	There was no effect of tocopherol on PD
	Parkinson Study Group (DATATOP study) [124]	800 untreated and early PD patients	Deprenyl 10 mg/d and/or tocopherol (vitamin E) 2000 IU/d	Alpha-tocopherol did not improve clinical features in patients with Parkinson's disease
	Vatassery et al. (DATATOP study) [83]	*n* = 18 (vitamin E group)/*n* = 5 (placebo group)	Tocopherol (vitamin E) 2000 IU/d	Treatment with vitamin E significantly increased the alpha-tocopherol concentrations in cerebrospinal fluid
	Taghizadeh et al. [125]	*n* = 30 (vitamin E group)/*n* = 30 (placebo group)	1000 mg omega-3 fatty acids plus 400 IU vitamin or placebo	Omega-3 and vitamin E cosupplementation in PD patients improved UPDRS compared with the placebo
Vitamin D	Suzuki et al. [113]	*n* = 56 (vitamin D3 group)/*n* = 58 placebo group)	Vitamin D3 1200 IU/d or placebo for 12 months	Vitamin D3 prevented the deterioration of the PD and especially patients with FokI TT genes
	Sato et al. [126]	*n* = 43 (vitamin D group)/*n* = 43 (placebo group)	1*α*(OH)D_3_ 1 *μ*g/d or placebo for 12 months	1alpha-hydroxyvitamin D3 supplements can reduce the risk of hip and other nonvertebral fractures in PD patients

**Table 3:** The basic study of vitamins in PD.

Vitamin	Authors	Object of study	Treatment	Conclusions
Vitamin B_3_ (nicotinamide)	Lu et al. [31]	Rotenone-PC12 cells	NMN (0.1 mM, 1.0 mM, 5 mM, and 10 mM) coculture	Attenuated apoptosis and improved energy metabolism
	Xu et al. [114]	MPTP-C57BL/6 mice	500 mg/kg/day for 5 days i.p.	Nicotinamide can alleviate MPTP-induced damage to dopaminergic neurons through antioxidant stress
	Jia et al. [115]	MPP(+)-SK-N-MC human neuroblastoma cells and alpha-synuclein transgenic Drosophila PD model	Nicotinamide concentration (21, 51, 101, 301, and 501 mg/L and 3, 15, 30, and 60 mg/100 g)	High doses of nicotinamide can reduce oxidative stress and improve mitochondrial function
	Anderson et al. [116]	MPTP-adult male C57Bl/6 mice	Nicotinamide (125, 250, or 500 mg/kg i.p.)	Recovered the striatal DA levels and SNc neurons after accepting nicotinamide
Vitamin C (ascorbic acid)	Khan et al. [117]	PD Drosophila model	L-Ascorbic acid (AA, 11.35 × 10^−5^ M, 22.71 × 10^−5^ M, 45.42 × 10^−5^ M, and 68.13 × 10^−5^ M for 21 days)	Except 11.35 × 10^−5^ M, other concentrations of AA attenuated the loss of climbing ability of PD model flies in a dose-dependent manner
	Yan et al. [52]	Mesencephalic precursors from the E12 rat	Ascorbic acid (0.1 *μ*M, 1 *μ*M, 10 *μ*M, 100 *μ*M, and 1 mM)	Ascorbic acid promoted the dopaminergic differentiation
	Seitz et al. [49]	Human neuroblastoma cell line SK-N-SH	Short-term incubation (100–500 *μ*M for 2 h) and long-term incubation (200 *μ*M for 5 days)	Ascorbic acid increased the DOPA production and tyrosine hydroxylase gene expression
	Pardo et al. [47]	Human neuroblastoma cell NB69	10^−3^ M ascorbic acid or 23 and 115 × 10^−3^ M alpha-tocopherol	Ascorbic acid prevents the levodopa toxicity and quinone formation, but alpha-tocopherol did not
	Sershen et al. [48]	MPTP-BALB/cBy mice	Ascorbic acid 100 mg/kg i.p.	Ascorbic acid may protect against the MPTP neurotoxicity
Vitamin E	Nakaso et al. [75]	MPTP-C57BL/6 mice	*δ*-Tocotrienol (100 *μ*g/kg for 4 days, p.o.)	*δ*-Tocotrienol administration inhibited the loss of dopaminergic neurons and improved the motor performance
	Sharma and Nehru [70]	Rotenone-Sprague-Dawley rats	Vitamin E (100 IU/kg/day for 35 days i.m.)	Vitamin E administration significantly improved locomotor activity and increased the dopamine level, GSH, and SOD
	Ortiz et al. [118]	MPTP-C57BL/6 mice	Vitamin E (50 mg/kg/day p.o.)	Vitamin E administration has decreased the COX-2 activity, LPO, and nitrite/nitrate level
	Pasbakhsh et al. [119]	6-OHDA-rat	Alpha-tocopherol acid succinate (24 IU/kg, i.m.)	Vitamin E treatment can protect locus coeruleus neurons in the PD model
	Roghani and Behzadi [69]	6-OHDA – Sprague-Dawley rats	d-*α*-Tocopheryl acid succinate (24 I.U./kg, i.m.)	Vitamin E treatment improved the rotational behaviour and prevented the reduction of tyrosine hydroxylase-immunoreactive cells
Vitamin D	Lima et al. [120]	6-OHDA-Wistar rats	1,25-(OH)_2_D_3_ (1 *μ*g/kg for 7 days or for 14 days, p.o.)	Vitamin D can protect the dopaminergic neurons by its anti-inflammatory and antioxidant properties
	Calvello et al. [121]	MPTP-male C57BL/6 N mice	Vitamin D (1 *μ*g/kg for 10 days, i.g.)	Vitamin D administration attenuates neuroinflammation and dopaminergic neurodegeneration
	Li et al. [122]	MPTP-C57BL/6 mice	Calcitriol (0.2, 1, and 5 *μ*g/kg/day for 7 days p.o.)	Calcitriol can significantly attenuate the neurotoxicity induced by MPTP
	Jang et al. [104]	Rotenone-SH-SY5Y cells	Calcitriol (0.0 *μ*M, 0.63 *μ*M, 1.25 *μ*M, 2.5 *μ*M, 5 *μ*M, and 10 *μ*M)	1,25-Dyhydroxyvitamin D_3_ can induce the autophagy to protect against the rotenone-induced neurotoxicity
	Cass et al. [123]	6-OHDA-male Fischer-344 rats	Calcitriol (0.3 or 1.0 *μ*g/kg/day for 8 days, i.h.)	Calcitriol can promote functional recovery of dopaminergic neurons and release of dopamine
	Sanchez et al. [100]	6-OHDA-Sprague-Dawley rats	1,25(OH)(2)D(3) (1 *μ*g/mL/kg/day for 7 days i.p.)	1,25(OH)(2)D(3) treatment increased the GDNF protein expression and partially restored TH expression
	Kim et al. [102]	6-OHDA Sprague-Dawley rats and MPTP-C57BL/6 mice	1,25-(OH)_2_D_3_ (1 *μ*g/mL at 1 mL/kg/day for 7 days, i.p.)	1,25-(OH)_2_D_3_ can inhibit the microglial activation and protect against nigrostriatal degeneration

LPO: lipid peroxides; COX-2: ciclooxigenase-2; TH: tyrosine hydroxylase; i.p.: intraperitoneal; i.m.: intramuscular; i.g.: intragastrical; i.h.: hypodermic injection; p.o.: peros.
